# A rare cause of spontaneous hemoperitoneum: intra-abdominal vein rupture

**DOI:** 10.2144/fsoa-2023-0029

**Published:** 2023-08-08

**Authors:** Moufida Mahmoudi, Ghada Gharbi, Amal Khsiba, Amine Jallouli, Asma Ben Mohamed, Manel Yakoubi, Mouna Medhioub, Lamine Hamzaoui, Khaled Bouzaidi, Mohamed Mousadek Azouz

**Affiliations:** 1 Department of Gastroenterology, Mohamed Taher Maamouri Hospital, Nabeul Tunisia; 2 Department of Radiology, Mohamed Taher Maamouri Hospital, Nabeul Tunisia

**Keywords:** ascites, cirrhosis, rupture of intra-abdominal vein, spontaneous hemoperitoneum

## Abstract

**Aim:**

Ruptures of the intra-abdominal vein causing a spontaneous hemoperitoneum in cirrhotic patients is a rare condition. However, diagnosis must be considered early in cirrhotic patients with hematic ascites as a delayed diagnosis with hemodynamic instability is associated with a poor prognosis.

**Case report:**

We present the case of a 54-year-old cirrhotic patient who presented a spontaneous hemoperitoneum due to the rupture of the intra-abdominal vein that was diagnosed during exploratory laparoscopy.

**Conclusion:**

Early diagnosis and management of spontaneous hemoperitoneum due to the rupture of intra-abdominal vein helps improve its prognosis.

## Plain language summary

Spontaneous hemoperitoneum is a rare but catastrophic condition caused by the rupture of the liver, spleen or abdominal vessels. In patients with cirrhosis, diagnosis of spotaneous hemoperitoneum is difficult. This is a case report of a 54-year-old cirrhotic man with a spontaneous hemoperitoneum due to the rupture of a vein within the abdomen. The diagnosis was suspected as the patient had signs of blood in the abdomen. To confirm spontaneous hemoperitoneum, the doctors performed a procedure called exploratory laparoscopy, which involves inserting a small camera into the abdomen. The laparoscopy confirmed the presence of blood and thus the diagnosis of spontaneous hemoperitoneum.

Portal hypertension, seen in patients with cirrhosis, often leads to collateralization of blood flow via variceal vessels that shunt blood from the portal to the systemic circulation, which can be the cause of paraumbilical vein permeabilization or intra-abdominal varices [1]. The rupture of these veins is uncommon. The most frequent circumstance of discovery is spontaneous hemoperitoneum. Prognosis is poor if the diagnosis is delayed [3–13].

We present the case of cirrhotic patient who presented with a spontaneous hemoperitoneum due to the rupture of the intra-abdominal vein. This was diagnosed during exploratory laparoscopy.

## Case presentation

A 54-year-old man with a history of hypertension treated with calcium channel blockers and steatohepatitis (NASH) cirrhosis was admitted to the gastroenterology department for oedemato-ascitic syndrome.

On examination, the patient had no fever or jaundice. Blood pressure was measured at 140/90 mmHg and heart rate at 68 bpm. He presented peripheral edema. Abdominal examination revealed collateral venous circulation with a grade three ascites. The biological findings showed anemia with hemoglobin at 10.2 g/dl. The platelet count was 112,000/mm^3^ and the prothrombin time was 53%. Liver tests revealed elevated transaminase levels with ALAT at 86 UI/l and ASAT at 98 UI/l. Total bilirubin blood level was 36 μmol/l and albuminemia 27 g/dl.

The ascites was hematic. It was a transudate with protein level at 10 g/l and cytological examination showed the presence of numerous red blood cells >10,000 elements.

**Figure 1.**
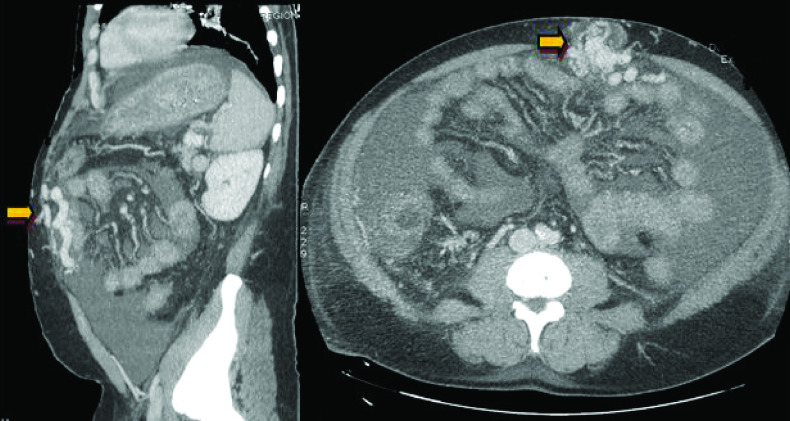
Computed tomography images showing a parietal varicose veins secondary to the permeabilization of the umbilical vein (yellow arrow).

As part of the etiological assessment of this spontaneous hemoperitoneum, an abdominal CT-scan was performed and showed the absence of active bleeding and absence of hepato-cellular carcinoma (HCC). However, a significant portal hypertension and collateral venous circulation was observed (Figure 1).

A diagnostic laparoscopy was performed which showed an important intra-abdominal and parietal venous circulation, more developed in the left abdominal wall but without active bleeding. This parietal circulation may be the cause of the hemoperitoneum as the bleeding could be intermittent.

Seven days after surgery, the patient presented with abdominal pain secondary to a fluid ascites infection. Antibiotic therapy with tazocillin and albumin perfusion was prescribed. Nevertheless, the patient presented with septic shock and multi-visceral failure. Unfortunately, the patient continued to present with septic shock and mutli-visceral failure and passed away 3 weeks later.

## Discussion

Spontaneous hemoperitoneum is defined as blood within the peritoneal cavity in the absence of a traumatic cause [1]. It is an uncommon condition, and fatal if treated inappropriately [2]. It is mostly caused by bleeding from tumors, gynecological causes and rupture of an intra-abdominal blood vessel in some vascular pathologies such as aneurysms and arteriovenous malformations.

When spontaneous hemoperitoneum occurs in patients with cirrhosis, we should firstly consider the diagnosis of hepatocellular carcinoma and intra-abdominal varices rupture [3].

The rupture of an intra-abdominal vein is rare. In fact, few case reports and series have been reported in the literature [3]. There are no meta-analyseis or systemic reviews which has limited epidemiological data [4,5]. Based on a surgical review, the umbilical vein is concerned in 20% of cases, Retzius veins in 17% and retroperitoneal varices in 14.3% [4,6].

Reviewing the cases that are previously reported in the literature, this condition seems to be associated with a significant mortality of 75%. This is due on one hand to a delayed diagnosis and on the other hand to the own comorbidities of cirrhotic patients [3,7].

The clinical presentation is nonspecific. The diagnosis is usually made in a cirrhotic patient with hemodynamic instability without external bleeding, a recent in-vascular gesture (puncture) or a significant abdominal traumatism [3,8]. In our case, the diagnosis was made early before alteration of hemodynamic parameters. The puncture of the ascites showed hematic fluid.

Diagnosis of this condition remains difficult. The role of the abdominopelvic scan in this case remains very limited. It can show the presence of an intra-peritoneal effusion and the absence of other causes of bleeding (splenic rupture or hemorrhage from hepatocellular carcinoma). Shanmuganathan *et al.* reported that attenuation values of active hemorrhage and clotted blood ranged from 85 to 370 HU (mean 132 HU) and 40 to 70 HU (mean 51 HU), respectively [2,9]. Interpretation of these results can sometimes be difficult for the radiologist, and this should not delay therapeutic management. Rarely, it allows to visualize venous bleeding from a varicose vein in the abdominal cavity [3,10]. In our case, the CT scan showed a significant portal hypertension and collateral venous circulation without active bleeding and the absence of other causes.

Data concerning the management of spontaneous hemoperitoneum due to the rupture of intra-abdominal vein are rare. There are no randomized trials due to the rareness of this situation. However, this condition seems to be underestimated, probably because most of the patients present tense ascites and terminal stage hepatic disease leading rapidly to death [6].

Reported treatment approaches include local umbilical vein ligation, embolization of paraumbilical veins, transjugular intrahepatic portosystemic shunt (TIPS), and laparoscopic clipping of the intra-abdominal vein [11].

The role of arteriography is controversial. Some authors suggest that arteriography and embolization has proved to be an inefficient investigation for diagnosis and treatment of retroperitoneal bleeding varices, it postpones the surgical treatment [6,8]. Others consider that it has a fundamental role in the treatment of intraabdominal vein rupture [2,11,12].

The main treatment of hemoperitoneum due to intra-abdominal variceal bleeding is surgery especially in patients with hemorrhagic shock. Nevertheless, the surgical portosystemic shunt or the TIPS can be considered for selected patients [5,13]. These invasive procedures can lead to severe complications, particularly infectious ones. A rigorous asepsis can avoid those complications.

## Conclusion

Spontaneous hemoperitoneum due to rupture of an intra-abdominal varicose vein in cirrhotic patients is a rare condition associated with a significant mortality. An early diagnosis can improve prognosis as it depends on early surgical management.

**Summary points**

- Spontaneous hemoperitoneum due to rupture of an intra-abdominal varicose vein is a rare condition in cirrhotic patients with a poor prognosis.
- Diagnosis of spontenous hemoperitoneum must be considered in a cirrhotic patient with hematic ascites or in the case of hemodynamic instability without external bleeding.
- Early diagnosis and management significantly improves the prognosis.
